# Effects of Modified Clay on *Phaeocystis globosa* Growth and Colony Formation

**DOI:** 10.3390/ijerph181910163

**Published:** 2021-09-27

**Authors:** Xiangzheng Ren, Zhiming Yu, Lixia Qiu, Xihua Cao, Xiuxian Song

**Affiliations:** 1 CAS Key Laboratory of Marine Ecology and Environmental Sciences, Institute of Oceanology, Chinese Academy of Sciences, Qingdao 266071, China; renxiangzheng@qdio.ac.cn (X.R.); lxqiulxqiu@126.com (L.Q.); caoxh@qdio.ac.cn (X.C.); songxx@qdio.ac.cn (X.S.); 2 Laboratory for Marine Ecology and Environmental Science, Qingdao National Laboratory for Marine Science and Technology, Qingdao 266237, China; 3 University of Chinese Academy of Sciences, Beijing 100049, China; 4 Center for Ocean Mega-Science, Chinese Academy of Sciences, Qingdao 266071, China

**Keywords:** *Phaeocystis globosa*, solitary cell, colony, HABs control, modified clay

## Abstract

*Phaeocystis globosa* is a globally distributed harmful algal blooms (HABs) species dominated by the colonial morphotype, which presents dramatic environmental hazards and poses a threat to human health. Modified clay (MC) can effectively flocculate HAB organisms and prevent their subsequent growth, but the effects of MC on colony-dominated *P. globosa* blooms remain uncertain. In this paper, a series of removal and incubation experiments were conducted to investigate the growth, colony formation and colony development of *P. globosa* cells after treatment with MC. The results show that the density of colonies was higher at MC concentrations below 0.2 g/L compared to those in the control, indicating the role of *P. globosa* colonies in resistance to environmental stress. Concentrations of MC greater than 0.2 g/L could reduce the density of solitary cells and colonies, and the colony diameter and extracellular polysaccharide (EPS) content were also decreased. The adsorption of MC to dissolved inorganic phosphorus (DIP) and the cell damage caused by collision may be the main mechanisms underlying this phenomenon. These results elucidate that the treatment with an appropriate concentration of MC may provide an effective mitigation strategy for *P. globosa* blooms by preventing their growth and colony formation.

## 1. Introduction

*Phaeocystis globosa*, a globally distributed phytoplankton species, is not only one of the main causes of harmful algal blooms (HABs) but also plays an important role in regulating the global climate and the structure of the food web [1,2,3,4]. *P. globosa* has a complex polymorphic lifecycle, alternating between solitary cells and colonial morphotypes. Solitary cells possess two equal heterodynamic flagella and a short haptonema, with a cell diameter of 3–9 μm [5,6]. Colonies are composed of polysaccharides that can form a spherical matrix in which thousands of colonial cells are uniformly embedded, and are able to reach up to 3 cm in diameter [7,8,9].

The formation of colonies is of great biological and ecological importance for the lifecycle of *P. globosa*. The thin and tough matrix of these colonies provides a defence mechanism against predators, viruses and other unfavorable environmental factors [10,11,12,13,14]. Additionally, the colony can serve as a “granary” for the storage of large amounts of organic matter and nutrients, which can provide energy for colonial cells in nutrient-limited environments [15,16]. Therefore, the formation of colonies contributes to the success of *P. globosa* in global marine systems, and *P. globosa* blooms are usually dominated by the colonial morphotype [3,17]. The most prominent features of *P. globosa* colonies are their high abundance, large size and high organic matter content, which cause particular damage during blooms. *P. globosa* blooms can produce large amounts of biomass and mucus foam, which accumulate along the coast and cause serious ecological hazards [15,18,19]. Additionally, it has been found that colonies can block fishing nets and interfere with important coastal infrastructures (such as nuclear power plants) due to their large diameter, posing a serious threat to economic development and human safety [20,21]. Because of the particular structure and important function of *P. globosa* colonies, the issue of how to remove them efficiently in emergencies and inhibit their re-formation has received extensive attention.

Modified clay (MC) is an effective method for HABs mitigation, with the advantages of an abundance of raw materials, low costs, high efficiency and non-pollution. Thus, this material has been widely studied and applied worldwide [21,22,23,24,25,26]. The principle of controlling HABs using MC can be summarized as direct effects and indirect effects. The direct effect involves settling HAB organisms from the surface to the bottom through flocculation, causing the cells to gradually die. The flocculation process is completed within 3 h, and the removal efficiency of HAB organisms can exceed 90% [21,24,27,28]. However, based on previous field experience, a removal efficiency of 70–80% is sufficient to control HABs, and the algal cells in the upper water do not regrow or contribute to a second bloom. Laboratory investigations on *Aureococcus anophagefferens* and *Karenia mikimotoi* showed that MC can disrupt the normal physiological processes in cells by inducing oxidative stress, inhibiting photosynthesis or causing other damage [29,30,31]. Moreover, water quality changes (such as the adsorption of nutrients and the effect of “mutual shading”) caused by MC were also explained by the inhibited growth of residual cells [21]. It can be concluded that MC can directly remove HAB organisms via flocculation and inhibit the growth of residual cells through indirect effects. As an effective method of HAB control, the use of MC has been successfully applied in many types of HAB mitigation [21,32,33].

Although previous studies on HAB mitigation using MC focused on HABs formed by unicellular microalgae, only a few studies have been conducted on *P. globosa* blooms. Cao et al. found that MC could effectively flocculate and settle *P. globosa* solitary cells and colonies [20], but the effects of MC on *P. globosa* cell growth and colony formation remained unclear. As previously mentioned, *P. globosa* tends to form colonies in resistance to environmental stresses [34,35], and MC presented indirect effects in controlling the growth of HABs [29,30,31]. Therefore, we assumed that the growth and colony formation of the remaining cells might be inhibited after MC treatment, which could potentially prevent the *P. globosa* blooms. To verify this hypothesis, in this study, cells treated with various concentrations of MC were further cultured to observe their growth, colony formation and colony development; moreover, the content of bound extracellular polysaccharide (bEPS) was used to represent the colonial matrix, so this was also evaluated. Changes in water quality factors, such as the pH and nutrient content, were simultaneously observed. Based on these results, the effects of various concentrations of MC on *P. globosa* cell growth and colony formation, as well as the potential mechanisms underlying these effects, were identified.

## 2. Materials and Methods

### 2.1. Experimental Materials

In this study, *P. globosa* was cultured at the Key Laboratory of Marine Ecology and Environmental Science, Institute of Oceanology, Chinese Academy of Sciences. Natural seawater was collected offshore in Qingdao, China, filtered through a 0.45-μm mixed-fiber membrane and sterilized by heating at 121 °C for 30 min. Subsequently, L1 medium without Na_2_SiO_3_ [36] was added to the seawater. The cultures were grown at 20 ± 1 °C with a 12-h light:12-h dark cycle and a light intensity of 60–70 μmol photons/(m^2^·s).

The MC used in this study was composed of natural clay and polyaluminum chloride (PAC). The natural clay was kaolin collected from Beihai, Guangxi Province, China. They were diluted in filtered seawater with a ratio of clay:PAC = 5:1 to produce a stock solution of 40 g/L; the stock solution was not mixed until it was required for the experiment [28,37].

### 2.2. Experimental Design

To avoid any possible interference of colony debris in the measurement of biomass and colony diameter, MC was added to *P. globosa* solitary cell culture in this study. Before the experiment, the *P. globosa* was incubated to the mid-exponential phase and passed through a 20-µm nylon sieve under gravity to obtain a solitary cell culture. The solitary cell culture was diluted with sterile seawater supplemented with L1 medium (without Na_2_SiO_3_) to an initial cell density of approximately 2000 cells/mL. Then, a given volume of diluted culture was placed in a beaker. Next, the MC stock solution (40 g/L) was added to obtain final MC concentrations in the cultures of 0 g/L (control group), 0.025 g/L, 0.05 g/L, 0.075 g/L, 0.1 g/L, 0.15 g/L, 0.2 g/L, 0.3 g/L and 0.5 g/L. Previous studies have confirmed that HAB organisms subjected to flocculation and settlement by MC will gradually die, and the organic matter in the flocs is effectively blocked and sealed, which will not impact algal cell growth in the medium [24,38,39]. Therefore, in this study, the sediments were discarded after the MC was fully settled in order to eliminate the potential impacts of flocs on sampling. After settlement for 3 h (removal efficiencies of the experimental groups reached 26 ± 6%, 40 ± 2%, 54 ± 16%, 55 ± 15%, 60 ± 5%, 73 ± 7%, 80 ± 1% and 90 ± 2%, respectively), the upper portion of the media (all culture above 3 cm from the bottom) with residual cells was transferred carefully to new 75-cm^2^ disposable cell culture bottles for further study. In this experiment, each group was treated in triplicate.

In the control group and the 0.1 g/L, 0.2 g/L and 0.5 g/L MC-treated groups, samples were taken every two days to determine the solitary cell density, colony density, colony size and various water quality parameters, such as the pH and nutrient content, and other samples were taken on the 10th day after MC was added to determine the cell abundance per colony and polysaccharide content. In the other MC-treated groups, the solitary cell density, colony density and colony size were measured only on the 10th day after MC was added because the *P. globosa* colony density and colony size reached their highest values on the 10th day in a preliminary experiment. The inhibition rates of MC on the growth and colony formation of solitary *P. globosa* cells were also calculated as follows:

(1) $$\mu =\frac{\left(N_{C}-N_{P}\right)}{N_{C}}\times 100\%$$

where *μ* is the inhibition rate, *N_P_* is the solitary cell or colony density in the MC-treated group and *N_C_* is the solitary cell or colony density in the control group on the same day.

### 2.3. Sampling and Analysis

#### 2.3.1. Microscopy

The tissue culture flasks were gently swirled immediately to obtain an even distribution of cells and colonies before sampling. Samples for microscopic measurement of cell abundances, colony abundances, colony diameter and cell number per colony were preserved in Lugol’s solution (final concentration of 2%) and measured under an inverted microscope (IX71, Olympus, Tokyo, Japan). The solitary cell density was measured using a 100-μL counting chamber [11]. Aliquots of known volume were pipetted from each group into a 24-well culture plate. After the colonies had settled completely, the colony density, colony size and cells per colony were measured using an inverted microscope with a calibrated micro-ruler. Colony diameter was estimated according to Wang et al. [40], where 30 representative colonies were measured in each sample; if there were fewer than 30 colonies in the sample, all of the colonies were measured. To explore the relationship between colonial cell density and colony size, as many colonies as possible were placed under an inverted microscope for quantification of colony size and colonial cell abundance. Total cell abundance in the colony was calculated by multiplying the cell abundance per unit area and surface area of the colony, and the colony diameter was calculated as an equivalent circular diameter. The abundance of colonial cells was calculated from colony abundance, colony diameter and the corresponding regression relationship between colonial cell density and colony size. All microscopic samples were counted within 24 h of collection in order to limit cell and colony disruption during preservation.

##### 2.3.2. pH and Nutrient Analysis

The pH value was measured using a pH meter (S400 Seven Excellence, Mettler Toledo, Zurich, Switzerland) calibrated with a standard buffer solution.

Samples taken for nutrient analysis were collected by filtration through 47-mm Whatman GF/F filters, and the filtrate was stored at −20 °C in sterile 50-mL centrifuge tubes until analysis. The nutrient contents (NO_3_^+^, NO_2_^+^, NH_4_^+^ and PO_4_^+^) were measured using a SKALAR Flow Analyzer (SKALAR Flow Analyzer, Skalar Ltd., Breda, Netherlands). The NO_3_^+^, NO_2_^+^ and NH_4_^+^ contents were quantified to determine the dissolved inorganic nitrogen (DIN), and the PO_4_^+^ content was measured to calculate the dissolved inorganic phosphorus (DIP).

#### 2.3.3. Polysaccharide

The polysaccharide fractions were divided into soluble extracellular polysaccharide (sEPS), bound extracellular polysaccharide (bEPS) and intracellular polysaccharide (IPS); with sEPS dissolved in the culture, while bEPS was used to represent the colonial matrix. The sEPS, bEPS and IPS contents were measured as described by Yang et al. and Li et al. with slight modifications [41,42]. Culture samples (10 mL) from each group were centrifuged at 11,000× *g* for 10 min at 4 °C, the precipitate was discarded, and then a triple volume of anhydrous ethanol was added to the supernatants. The supernatants were settled for at least 24 h and then centrifuged at 12,000× *g* for 10 min at 4 °C. The precipitates were used to assay the sEPS content. An additional 10 mL of culture sample was collected from each group, the pH was adjusted to 10 with NaOH, and the samples were incubated in 45 °C water for 4 h to extract the bEPS. After cooling, the collections were centrifuged at 11,000× *g* for 10 min at 4 °C, and the supernatants were purified and settled as described above and were used to measure the extracellular polysaccharide (EPS) content (the EPS content was the sum of the sEPS and bEPS contents). Ten mL of the culture was collected in a centrifuge tube and subsequently sonicated for 3 min to break up the cells and then placed in 100 °C water and incubated for 1 h. After cooling, the samples were centrifuged, purified and settled as described above and were used to measure the total polysaccharide (TPS) content (the TPS content was the sum of the EPS and IPS contents). The polysaccharide samples extracted according to the above method were fully dissolved in ultrapure water and then filtered through 0.45 mm pore-size syringe filters (Millipore, Germany). The filtrates were used to measure polysaccharide content by the phenol-sulfuric acid method and calibrated by glucose solutions. The bEPS content and IPS content were obtained by subtraction.

### 2.4. Statistical Analysis

All data are shown as the mean values ± standard deviation (SD). Statistical analysis was performed using SPSS software (version 19, IBM Corp., Armonk, NY, USA), and the significance level for all statistical tests was set to *p* = 0.05. The differences in the parameters between each pair of groups were assessed by one-way analysis of variance (one-way ANOVA). Linear regression fitting was performed for log cells per colony and log colony diameter using Origin (version 9.0, Origin Lab, Northampton, MA, USA), and the slopes of pairs of groups were compared by one-way analysis of covariance (one-way ANOCVA). The figures in this paper were created using Origin 9.0.

## 3. Results

### 3.1. Solitary Cell Abundances

**Figure 1:**
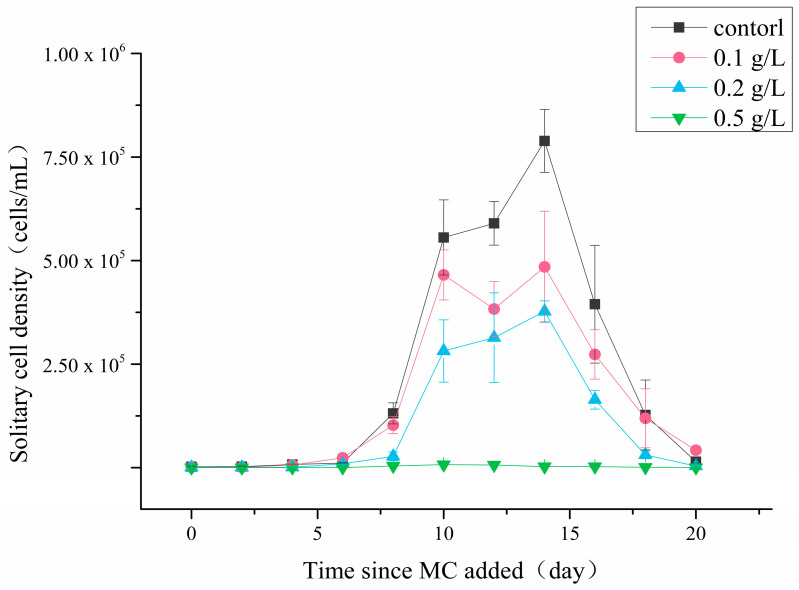
Changes in the density of *P. globosa* solitary cells after treatment with different concentrations of MC.

The solitary cell abundances of different experiment systems reduced with MC concentration improved after MC treatment (Figure 1). In the control group, the *P. globosa* solitary cells entered the rapid growth stage after a 4-day lag phase, then reached the highest abundance of 7.2 × 10^5^ cells/mL on the 14th day. In the 0.1 g/L and 0.2 g/L MC-treated groups, the solitary cells exhibited similar growth trends and began to grow rapidly on the 4th day and 6th day after MC was added, reaching only 4.5 × 10^5^ cells/mL and 3.8 × 10^5^ cells/mL, respectively. The solitary cell density of the 0.5 g/L MC-treated group remained at a low level throughout the experiment, without a significant increase, and the maximum density was only 6.4 × 10^3^ cells/mL. The experimental results showed that MC could inhibit the growth of solitary cells.

**Figure 2:**
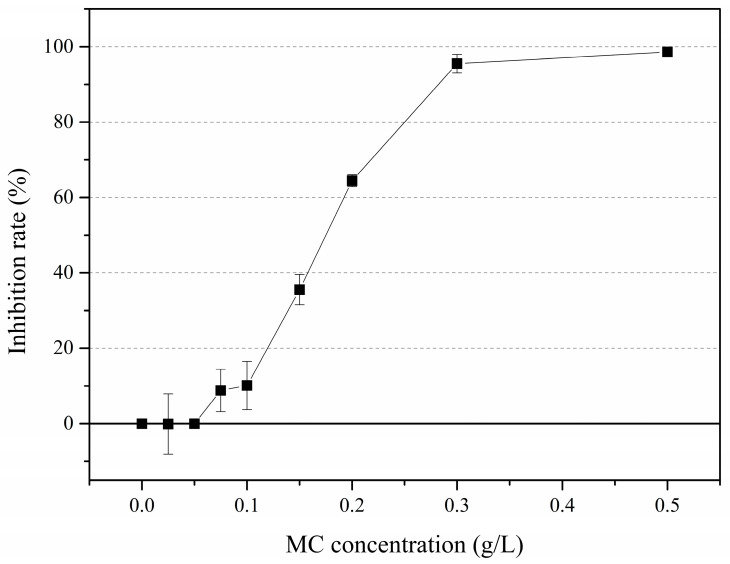
Inhibition rates of the growth of *P. globosa* solitary cells on the 10th day under different MC concentrations.

The inhibition rates on the 10th were used to evaluate the effects that different MC concentrations exerted on the growth of the remaining solitary cells (Figure 2). When treated by 0.025 g/L and 0.05 g/L MC, inhibition rates were both lower than 1%, which indicated a negligible inhibition. MC restricted the growth of solitary cells when concentrations were higher than 0.075 g/L. The inhibition rate gradually increased as the MC concentration increased and had reached 96% in the 0.3 g/L MC treatment.

### 3.2. Colony Abundances

**Figure 3:**
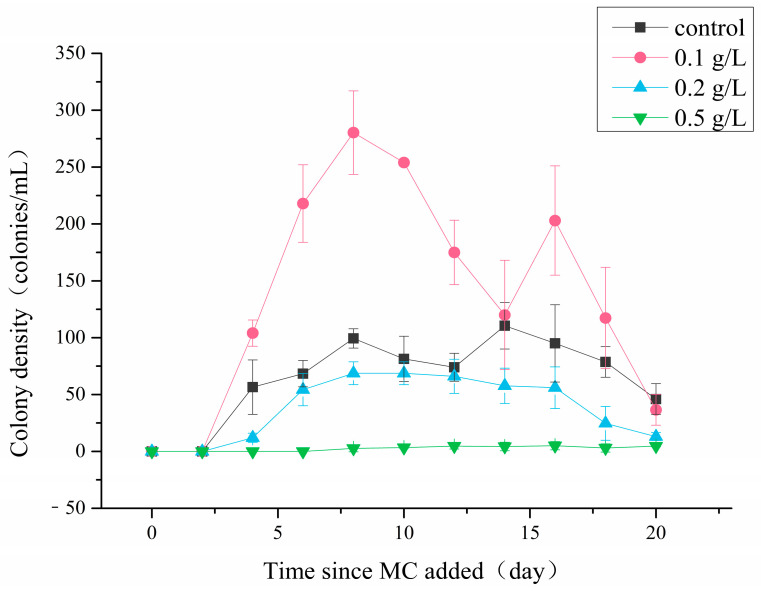
Changes in the density of *P. globosa* colonies after treatment with different concentrations of MC.

The *P. globosa* colonies abundances varied after treated by different concentrations of MC (Figure 3). Colonies were first observed on the 4th day in the control group and the 0.1 g/L, 0.2 g/L MC-treated groups, then their densities increased rapidly. In the 0.5 g/L MC-treated group, colonies were first observed on the 8th day, and the density remained low (<10 colonies/mL) during the incubation. On the 8th day of the incubation, the colony density in the 0.1 g/L MC-treated group reached the maximum of 280 colonies/mL, while that in the control group was only 99 colonies/mL. The *P. globosa* colony density in the 0.1 g/L MC-treated group increased again on the 14th day and reached 229 colonies/mL, in which a large number of small colonies were observed. The colony densities of the 0.2 g/L and the 0.5 g/L MC-treated groups were significantly lower than that of the control group (*p* < 0.05), and the maxima of these two groups were 68 colonies/mL and 5 colonies/mL, respectively.

**Figure 4:**
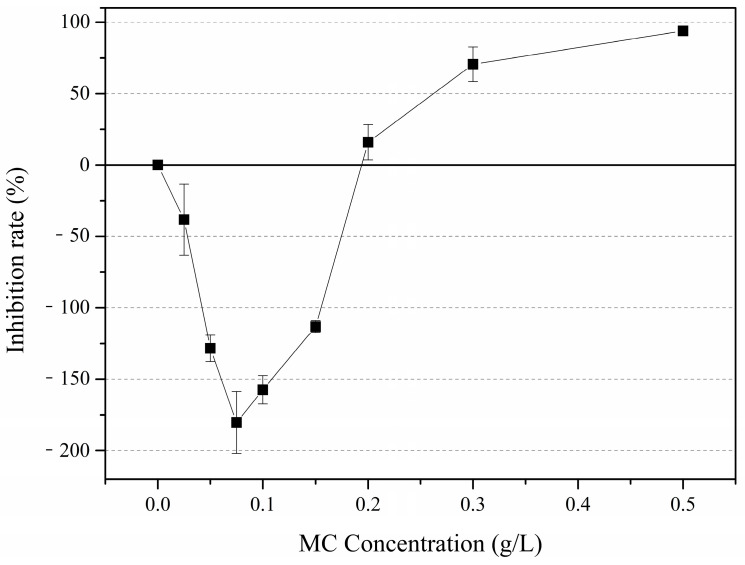
Inhibition rates of *P. globosa* colony formation on the 10th day under different MC concentrations.

To further verify the relationship between MC concentration and *P. globosa* colony formation, we explored the inhibition rates on the 10th day of colony formation (Figure 4). The colony formation was promoted under low concentrations of MC (<0.2 g/L), and the most obvious promoting effect was obtained in the 0.075 g/L MC-treated group, of which the colony density was 2.8-fold higher than that of the control group. When the concentration of MC was higher than 0.2 g/L, the colony formation was inhibited, and the inhibition rates were 15%, 75% and 96% under MC concentrations of 0.2 g/L, 0.3 g/L and 0.5 g/L, respectively.

### 3.3. Colony Morphology

**Figure 5:**
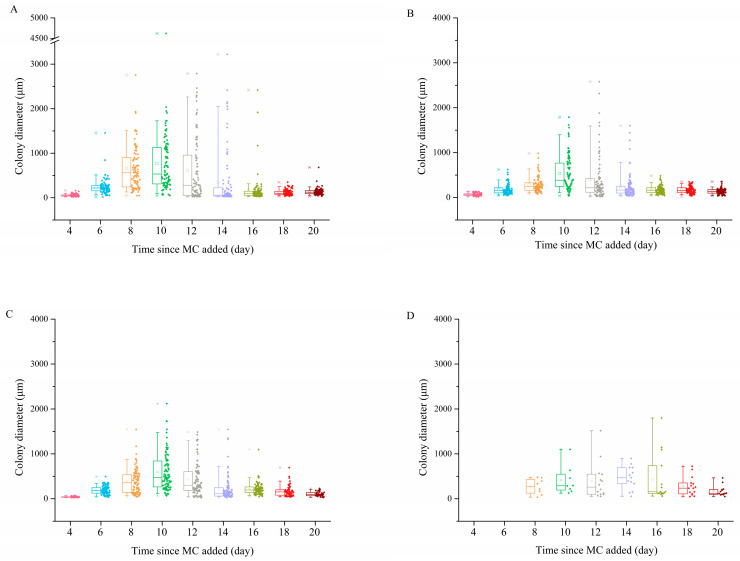
Changes in *P. globosa* colony diameter in the (**A**) control group, (**B**) 0.1 g/L MC-treated group, (**C**) 0.2 g/L MC-treated group and (**D**) 0.5 g/L MC-treated group. The error bars of the 5% and 95% are shown together below and above the box. Square dots in the box represent the mean values and the middle lines represent the median.

To analyze the colony morphology, variations of *P. globosa* colony diameters in each group were described (Figure 5). For all the groups, the mean diameters of the colonies initially increased and then decreased with increasing incubation time, and the highest mean values were detected on the 10th day. In addition to the variation with time, ANOVA revealed that the colony diameters in the MC-treated groups were significantly smaller than those in the control group (*p* < 0.01), and the mean colony diameter was negatively correlated with the MC concentration. In the control group, the colony diameter ranged from 50 to 4620 μm, with a mean of 672 μm (n = 90). The mean colony diameters in the 0.1 g/L, 0.2 g/L and 0.5 g/L MC-treated groups were 535 μm (n = 90), 511 μm (n = 90) and 411 μm (n = 8), respectively. Additionally, the largest colony in the control group was observed on the 10th day with a diameter of 4620 μm, whereas that in the MC-treated groups was observed on the 12th day in the 0.1 g/L MC-treated group with a diameter of only 2580 μm.

**Figure 6:**
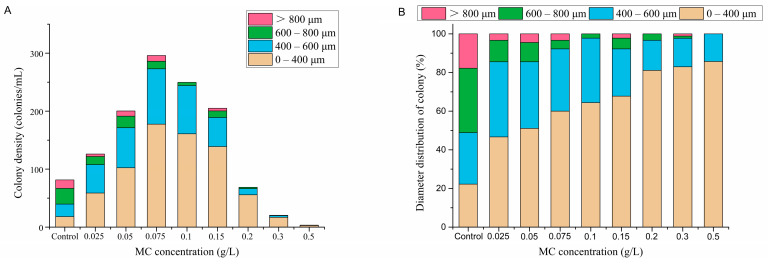
(**A**) Density and (**B**) percentage of the colonies in different ranges divided by colony diameter on the 10th day after MC addition.

Colony densities in different diameter ranges on the 10th day were further analyzed by groups. It was found that treatment with all eight concentrations of MC reduced the abundance of large colonies (>600 μm) (Figure 6A), even though the total colony abundance increased in some MC-treated groups. The colony density and the colony diameter were reduced in the groups treated with MC concentrations of 0.2 g/L, 0.3 g/L and 0.5 g/L, and large colonies (>600 μm) were rarely or never observed. The above phenomenon was also reflected in the distribution of colony diameters (Figure 6B). In the control group, more than 51% of the colonies were larger than 600 μm, and only 22% of the colonies were smaller than 400 μm. In the MC-treated groups, the above percentages changed to less than 20% and more than 45%, respectively, and the proportion of large colonies (>600 μm) decreased with increasing MC concentration. Taken together, these results suggest that there is an association between MC treatment and the decrease in *P. globosa* colony size.

**Figure 7:**
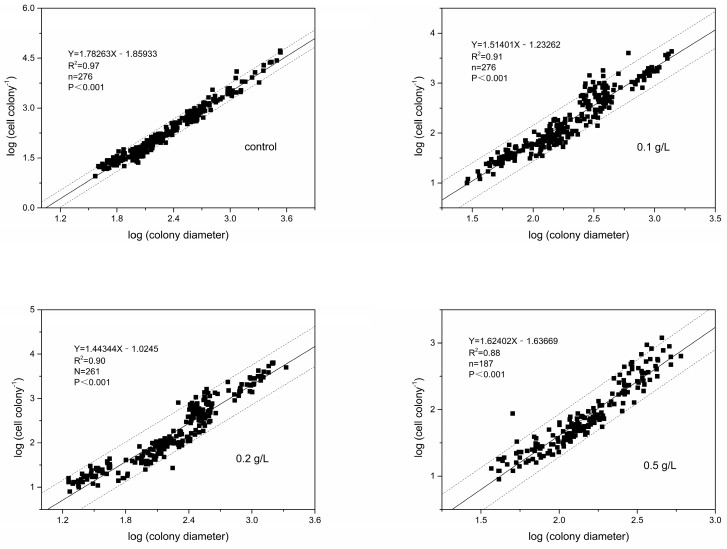
Relationship between the log cell number per colony and log colony diameter. (**a**) control group, (**b**) 0.1 g/L MC-treated group, (**c**) 0.2 g/L MC-treated group and (**d**) 0.5 g/L MC-treated group. Solid lines are linear regressions and dotted lines represent 95% confidence intervals, and n represents the number of colonies.

In all groups, there was a significant linear log-log relationship between the cell number per colony and colony diameter (*p* < 0.001), with slope values of 1.78 (control group), 1.51 (0.1 g/L group), 1.44 (0.2 g/L group) and 1.62 (0.5 g/L group) (Figure 7). The slope of the regression is less than 2, indicating that the cell abundance per unit colony surface area decreased as the colony diameter increased. In addition, the slope of the regression in the MC-treated group was significantly smaller than that in the control group (*p* < 0.01), which indicates that the colonies had fewer cells per unit colony surface area after MC treatment.

### 3.4. Polysaccharide Contents

**Figure 8:**
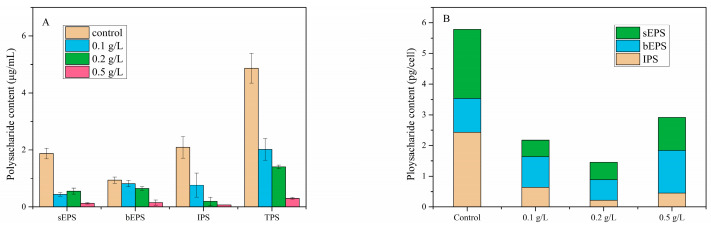
(**A**) Polysaccharide content and (**B**) polysaccharide fraction per cell on 10th day after various concentrations of MC added.

The TPS, sEPS, bEPS and IPS contents in each group on the 10th day were shown in Figure 8A. MC treatment strongly influenced the contents of sEPS, IPS and TPS in the cultures, which were more than 50% lower than those in the control group under 0.1 g/L, 0.2 g/L and 0.5 g/L MC treatment (*p* < 0.01). The bEPS was the main component of the colonial matrix and was thus given particular attention here. In our study, there was no significant difference in bEPS content among the control group, 0.1 g/L MC-treated group and 0.2 g/L MC-treated group. However, the bEPS content was significantly reduced under 0.5 g/L MC (*p* < 0.01), with only 16% of that in the control group.

The TPS, sEPS, bEPS and IPS contents per cell were calculated by the corresponding cell abundances (Figure 8B). The TPS contents per cell were significantly reduced from 5.78 pg/cell to 2.17 pg/cell (0.1 g/L group), 1.45 pg/cell (0.2 g/L group) and 2.89 pg/cell (0.5 g/L group), respectively. However, the proportions of bEPS to TPS were increased from 19% to 46%, 46% and 48% under the 0.1 g/L, 0.2 g/L and 0.5 g/L MC treatments, respectively, and the bEPS contents per cell were not significantly changed.

### 3.5. Environmental Parameters

**Figure 9:**
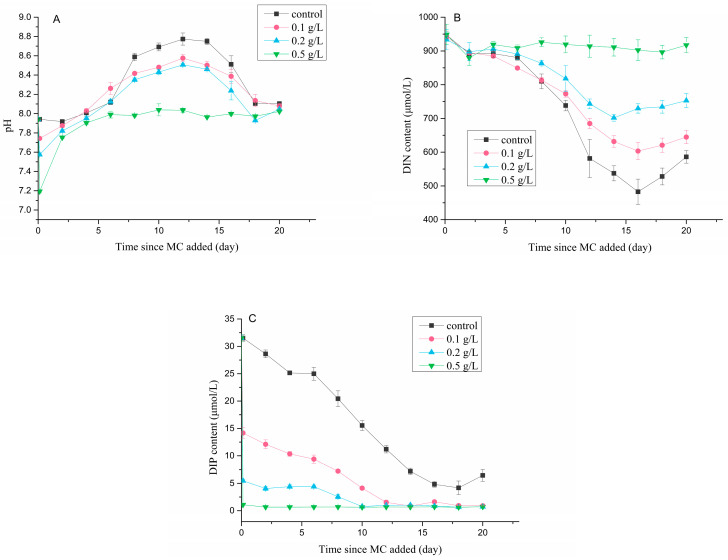
Changes in (**A**) pH, (**B**) DIN content and (**C**) DIP content in the *P. globosa* culture after MC treatment.

The pH slightly decreased with increasing MC concentrations: treatment with 0.1 g/L, 0.2 g/L and 0.5 g/L MC for 3 h decreased the pH from 7.9 to 7.7, 7.6 and 7.2, respectively (Figure 9A). However, the pH in the MC-treated groups recovered rapidly after sediment removal, and the value was very close to that of the control group on the 4th day after MC was added. Subsequently, the pH in the 0.5 g/L MC-treated group remained relatively stable, and the pH in the other groups tended to initially increase and then decrease with incubation.

The DIN contents did not decrease after MC treatment for 3 h (Figure 9B). In the control group, the 0.1 g/L and 0.2 g/L MC-treated groups, the DIN contents decreased gradually with *P. globosa* growth, while that in the 0.5 g/L MC-treated group did not change significantly. The minimum DIN contents in each group increased with increasing MC concentrations, with values of 467 μmol/L (control), 592 μmol/L (0.1 g/L group), 691 μmol/L (0.2 g/L group) and 873 μmol/L (0.5 g/L group), respectively. After approximately 16 days, the DIN content in each group increased slightly, but it was still significantly lower than the initial content.

The DIP contents were significantly reduced by treatment with MC for 3 h (Figure 9C), where 0.1 g/L, 0.2 g/L and 0.5 g/L MC reduced the DIP contents from 31.51 μmol/L to 14.17 μmol/L, 5.47 μmol/L and 1.09 μmol/L, respectively (corresponding to 45%, 17% and 3% of the initial content, respectively). The DIP content of the control group decreased with the increasing incubation time and reached the minimum value of 4.19 μmol/L on the 18th day, whereas the DIP contents in the MC-treated groups decreased to 1.00 μmol/L on the 14th day (0.1 g/L group), 10th day (0.2 g/L group) and 2nd day (0.5 g/L group) and remained stable for the rest of the experiment. In general, MC treatment significantly reduced the DIP content, and the DIP content in the MC-treated groups was gradually exhausted with increasing incubation time, while it remained sufficient in the control group.

## 4. Discussion

### 4.1. Effects of MC on P. globosa Solitary Cell Abundances

In this study, treatment with MC effectively removed *P. globosa* solitary cells, and the removal efficiency was positively correlated with the MC concentration. The main mechanism underlying the mitigation of HABs by MC is the settlement of HAB organisms through flocculation [21,28]. Higher MC concentrations therefore increased the possibility of collision and flocculation between the clay particles and *P. globosa* cells and thus contributed to a higher removal efficiency. The removal efficiency exceeded 80% after 0.3 g/L MC treatment, which was consistent with previous findings [21,43].

Notably, uncleared cells (those remaining in the upper layer after the MC treatment) were the object of interest in this paper since they might suffer indirect effects as a result of the MC. Zhu et al. found that residual cells after MC treatment showed deformation, irregular depressions and other cell damage [44]. Evidence from physio-biochemistry and transcriptome analyses has also shown that MC treatment can induce reactive oxygen species (ROS) in residual cells, which leads to oxidative damage and disrupted physiological processes, and inhibited their growth [29,30]. In this study, based on the changes in *P. globosa* solitary cell abundances, it was apparent that treatment with MC could inhibit *P. globosa* growth, and the inhibition efficiency was positively correlated with the MC concentrations. These results were consistent with previous studies on *Aureococcus anophagefferens* and *Karenia mikimotoi* [29,31].

**Figure 10:**
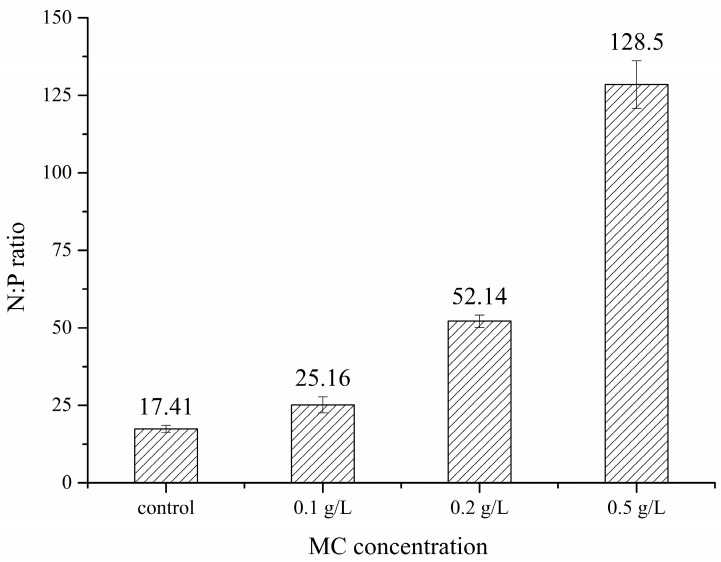
The absorption ratios of DIN and DIP during the incubation of *P. globosa*.

Treatment with MC dose-dependently inhibited the growth of solitary cells, which could be attributed to the following two reasons. Firstly, as mentioned above, collision and other interactions between clay particles and cells could cause serious damage in cells [29,30,31,44]. The collision frequency between the clay particles and *P. globosa* cells increased with increasing MC concentration, and the percentage of damaged cells in the residual cells also increased [21]. The cell damage caused by collisions might be one of the main factors that contributes to the reduction in *P. globosa* solitary cell abundance. Second, MC could affect HAB organisms’ growth by changing the environment [21]. In this study, treatment with MC slightly affected the DIN contents and pH, which indicated that DIN and pH were not key factors affecting *P. globosa* biomass. However, MC could effectively adsorb DIP in the medium, and the adsorption efficiency could exceed 97%. The absorption ratios of DIN and DIP during the incubation of *P. globosa* were calculated by the comparison between the reduction in the amounts of DIN and DIP (Figure 10). A previous study proved that N:P ratios greater than 35 represented a P-limitation [45], and photosynthesis efficiency reduced due to decreased synthesis of ATP under P-limitation [46,47]. The absorption ratios of DIN:DIP were much higher than 35 during treatment with 0.2 g/L and 0.5 g/L MC, indicating that *P. globosa* cells in these groups were under P-limitation, which may have affected *P. globosa* cell growth.

### 4.2. Effects of MC on P. globosa Colony Abundances and Colony Morphology

The *P. globosa* bloom is dominated by colonial morphotype, which is the most prominent feature of *P. globosa* and responsible for many unique environmental problem [3,4,48]. Therefore, the changes in colony abundances after MC treatment were the focus of our attention. In contrast to the changes in solitary cell abundances, the densities of colonies under low concentrations (<0.2 g/L) of MC treatment were higher than those of the control group, suggesting that MC stimulated colony formation at these concentrations. When the MC concentration was increased to 0.2 g/L or higher, it inhibited colony formation, and the inhibition rate reached 95% at an MC concentration of 0.5 g/L. These results indicated that MC at an appropriate concentration could effectively inhibit *P. globosa* colony formation and prevent *P. globosa* bloom recurrence.

Interestingly, treatment with a low concentration of MC (<0.2 g/L) increased the abundance of *P. globosa* colonies, which might be a potential risk of using MC to control *P. globosa* blooms. However, the abundances of total cells, solitary cells and colonial cells on the 10th day after MC addition were estimated and are shown in Table 1, and the results showed that treatment with low concentrations of MC reduced the biomass of *P. globosa*. The decrease in colonial diameter and polysaccharide content indicated that MC reduced the risk of mucus foam accumulation and net blockage. This result suggests that MC can control *P. globosa* blooms and reduced damage even at low concentrations.

An interesting finding was that the abundances of colonies increased after treatment with a low concentration of MC (<0.2 g/L), which was suspected to be caused by the role of *P. globosa* colonies in resistance to environmental stress. In general, the formation of colonies can be regarded as a defense mechanism in adverse environments, and environmental stresses can stimulate the formation of *P. globosa* colonies [34,35]. MC caused slight cell damage and environmental changes at low concentrations, which might have promoted colony formation in *P. globosa* residual cells. We also observed that the percentages of TPS accounted for by bEPS per cell were significantly increased. Polysaccharides consistently played a major role in the formation and maintenance of *P. globosa* colonies [9,49]; among them, the bEPS fraction could be used to determine the polysaccharide content in the colony matrix. This indicated that more energy had to be directed to form bEPS and maintain the stability of the colony structure in *P. globosa* cells. In summary, *P. globosa* tended to form colonies to improve its resistance after treatment with MC.

In addition, *P. globosa* solitary cells need to undergo the following steps to form colonies: flagella loss, settlement to a surface, mucus secretion and colony formation [5,50,51]. Among these processes, the stable substrate contributes to colony matrix accumulation and plays an important role in colony formation [6,52,53]. Most of the clay particles had flocculated and settled to the bottom within 3 h of MC addition, but some tiny clay particles remained in the upper portion of the media, which could be observed with the naked eyes or a microscope. These residual clay particles could have provided a large number of substrates for *P. globosa*, which would have aided in colony formation.

The *P. globosa* colony formation was significantly inhibited after 0.3 g/L and 0.5 g/L MC treatment, and the inhibitory efficiency was positively correlated with the MC concentration, which might be related to the cell damage and environmental changes described above. According to previous studies, a new *P. globosa* colony developed from a single cell, and the colonial cell abundance was increased by mitosis [6,51]. Thus, it can be concluded that the colonies originate from cells with no damage or slight damage. Several other studies have shown that treatment with either oxidant or marine bacteria could induce cell damage in *P. globosa* cells and inhibited colony formation [43,54,55]. Treatment with a high concentration of MC increased the collision frequency and decreased the percentage of healthy cells in the residual cells; thus, the formation of colonies was inhibited. Additionally, MC treatment could reduce the nutrient level in water, especially the DIP content, and the growth of colonial cells was seriously restricted because colonial cells were at a disadvantage for absorbing nutrients [56,57]. Thus, the formation of colonies was inhibited after treatment with a high concentration of MC.

In summary, MC treatment exerted two contrasting effects on *P. globosa* solitary cells from colonies. MC could inhibit colony formation by causing cell damage and changing the environment. In contrast, tiny residual clay particles could be employed as a substrate for forming new colonies, thus leading to colony formation. In the groups treated with low concentrations of MC (<0.2 g/L), the inhibitory effect was not significant due to slight cell damage and environmental changes, and the promoting effect was dominant. As the concentration of MC increased, the inhibitory effect became stronger than the promoting effect, and thus, the colony density of the groups treated with a high concentration of MC (≥0.2 g/L) was lower than that of the control group.

The colony morphology results showed that colony development was inhibited regardless of the increase in colony abundance. Colony morphology has also been a focus of *P. globosa* bloom studies, with colony diameter being the most important feature. *P. globosa* colonies generally ranged from 30 to 3000 μm in diameter, and giant colonies outside this range were often observed [8,18,58]. In this study, more than 90% of the colonies were in the range of 0 to 3000 μm in diameter. The comparison among groups showed that MC decreased the colony size, indicating that the risk of blocking nets during blooms could be decreased.

Moreover, the linear relationship between log cell number per colony and log colony diameter in each group was fitted, and the slopes showed that the colonies had fewer cells per unit colony surface area after MC treatment. The abundances of total cells, solitary cells and colonial cells at the 10th day after MC addition were estimated by the formulas (Table 1). Contrary to our expectations, the proportion of colonial cells did not increase after MC treatment, even though the colony abundance increased. As mentioned above, treatment with MC promoted the formation of *P. globosa* colonies, but the proportion and density of colonial cells were reduced, and the colonial diameter was also decreased, indicating that the development of colonies was inhibited.

According to previous studies, *P. globosa* colonies originated from cells with no damage or slight damage, which indicated that cell damage caused by collision contributed little to the inhibition of colony development. The adsorption of DIP by MC might be the main mechanism underlying the inhibition of the development of *P. globosa* colonies. In the present study, the DIP contents in all the MC-treated groups declined steadily to below 1.0 μmol/L with incubation. Several studies have shown that phosphate was the primary factor affecting the development of *P. globosa* colonies [34,40], and the growth rate of *Phaeocystis* sp. colonial cells was significantly decreased when the DIP content was below 2.0 μmol/L [56]. The absorption ratios of DIN and DIP during the incubation of *P. globosa* also demonstrated that the *P. globosa* cells were subjected to P-limitation after MC treatment. Additionally, polysaccharides were the main components of *P. globosa* colonies, with glucose accounting for more than 85% of the total polysaccharides [9,47]. Therefore, colonial cells must produce large amounts of glucose through photosynthesis during colony development. Photosynthesis efficiency was reduced due to enhanced intracellular pH, a reduced Calvin cycle and decreased synthesis of ATP under P-limitation [46,47]. In this study, the TPS content per cell of *P. globosa* was reduced after treatment with MC, which also proved that polysaccharide synthesis was inhibited. Although the bEPS content per cell was still considerable after MC treatment, the total number of colonial cells was too low to form a large-scale colony because the ability of colonial cells to secrete polysaccharides had a cap. The DIP content was significantly reduced by MC, which inhibited the reproduction of *P. globosa* colonial cells and reduced the colony matrix, and thus inhibited the development of colonies.

The results of this study showed that MC not only exerted a satisfactory inhibitory effect on *P. globosa* solitary cell and colony density but also reduced the colony size and polysaccharide content; the study thus provides a references for *P. globosa* bloom mitigation using MC. However, additional research should be performed in order to validate the potential mechanism responsible for these effects. The growth and colony formation of *P. globosa* in the field were probably regulated by a combination of factors; therefore, determining whether MC can inhibit *P. globosa* growth and colony formation and prevent the recurrence of *P. globosa* blooms in the field require more comprehensive, larger-scale experiments and field observations.

## 5. Conclusions

In the present study, to identify factors that are involved in MC controlling *P. globosa* blooms except flocculation, the effects of MC on *P. globosa* solitary cell growth, colony formation and colony development were investigated. The results showed that (1) MC treatment could effectively remove *P. globosa* solitary cells and inhibit their growth, with the inhibition efficiency of the 0.3 g/L MC treatment exceeding 90%. (2) The colony abundances increased under MC concentrations of less than 0.2 g/L. When the MC concentration was higher than 0.2 g/L, the formation of *P. globosa* colonies was inhibited, and the inhibition efficiency under 0.5 g/L MC exceeded 90%. (3) MC could inhibit colony development and cause a decrease in colony size, colonial cell density and polysaccharide content. The cell damage and DIP content decreases caused by MC might represent the main mechanisms underlying these phenomena. These results indicate that an appropriate concentration of MC can effectively eliminate *P. globosa* blooms, inhibit the regrowth and colony formation of residual cells, limit the development of colonies and prevent the recurrence of *P. globosa* blooms.

## Figures and Tables

**Table 1:** Densities and percentages of total cell abundance for solitary cells and colonial cells at 10th day after addition of various concentrations of MC.

Groups	Solitary Cells		Colony Cells		Total Cells
	Density (×10^3^ Cell/mL)	Percentage (%)	Density (×10^3^ Cell/mL)	Percentage (%)	Density (×10^3^ Cell/mL)
Control	555.8 ± 90.6	65	300.8 ± 35.1	35	856.7 ± 157.3
0.1 g/L	465.56 ± 60.3	66	238.1 ± 51.3	34	703.6 ± 17.9
0.2 g/L	281.7 ± 75.2	66	143.0 ± 38.4	34	424.6 ± 57.9
0.5 g/L	7.6 ± 0.8	76	2.4 ± 0.8	24	10.0 ± 1.7

## Data Availability

Data are available upon request; please contact the contributing authors.
